# Supplementary material for: Bioinspired asymmetric amphiphilic surface for triboelectric enhanced efficient water harvesting
Source: Nat Commun. 2022 Jul 18;13:4168. doi: 10.1038/s41467-022-31987-w (PMC9293931; doi:10.1038/s41467-022-31987-w)
Supplement: Supplementary file 1 — Supplementary Information [file 41467_2022_31987_MOESM1_ESM.pdf]

Supplementary Information

Bioinspired asymmetric amphiphilic surface for triboelectric enhanced efficient water harvesting

*Song Zhang[1], Mingchao Chi[1], Jilong Mo[1], Tao Liu[1], Yanhua Liu[1], Qiu Fu[1], Jinlong Wang[1], Bin Luo[1], Ying Qin[1], Shuangfei Wang[1], Shuangxi Nie[1,] **

[1]School of Light Industry and Food Engineering, Guangxi University, Nanning 530004, PR China

*Corresponding author. Email: nieshuangxi@gxu.edu.cn (S. Nie)

Supplementary Figure 1-22

Supplementary Table 1-2

Supplementary Note 1. Calculation of the water harvesting efficiency

The water harvesting efficiency (WHE) in our manuscript was calculated by Equation S1,

$$WHE = \frac{m}{At} \tag{S1}$$

Where m is the mass of collected water, A is the area of the collector, and t is the harvesting time. In the manuscript, the WHE of asymmetric amphiphilic surface was calculated using the projected area A_1. According to the editor's suggestion, we performed calculations using the rectangular area A_2 occupied by the asymmetric amphiphilic surface and obtained a highest WHE of 24.08 kg/m^2 h (Fig. S1).

In addition, water harvesting efficiency per mass ($WHEm$) was calculated by Equation S2:

$$WHE_m = \frac{m_1}{m_2 t} \tag{S2}$$

Where m_1 is the mass of the collected water, m_2 is the mass of the asymmetric amphiphilic surface, and t is the harvesting time. After calculation, related $WHEm$ was 304.99 g/g h.

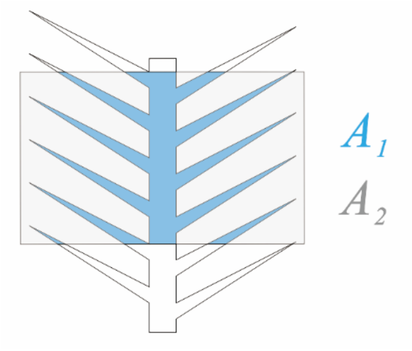

Supplementary Figure 1. The projected and occupied area of the amphiphilic asymmetric surface when calculating the water harvesting efficiency.

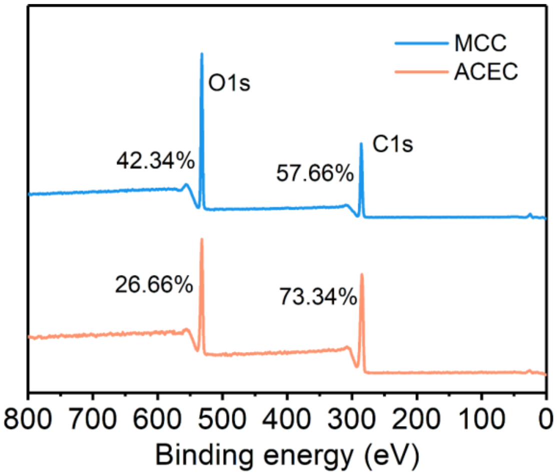

Supplementary Figure 2. XPS spectrum of the MCC and ACEC.

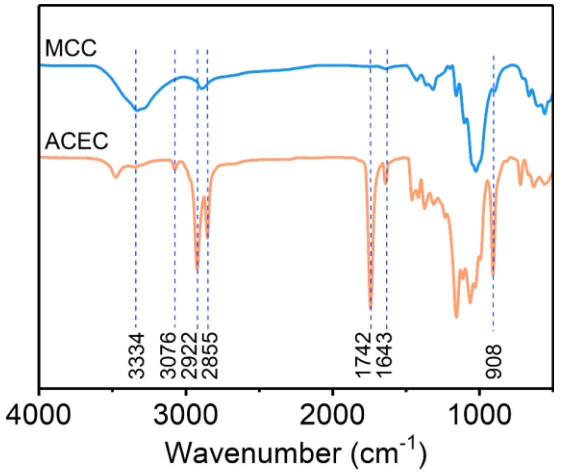

Supplementary Figure 3. FTIR spectra of the MCC and ACEC.

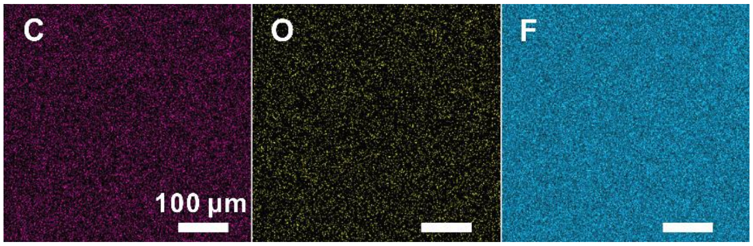

Supplementary Figure 4. EDX-mapping images of C, O and F element of FEP.

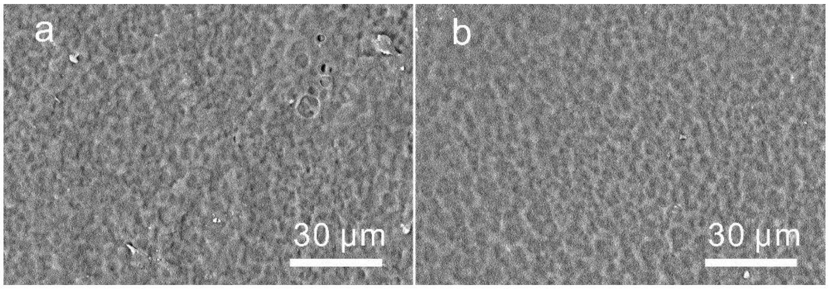

Supplementary Figure 5. a, b SEM images of ACEC@FEP and after the water droplets sliding down (10,000 times).

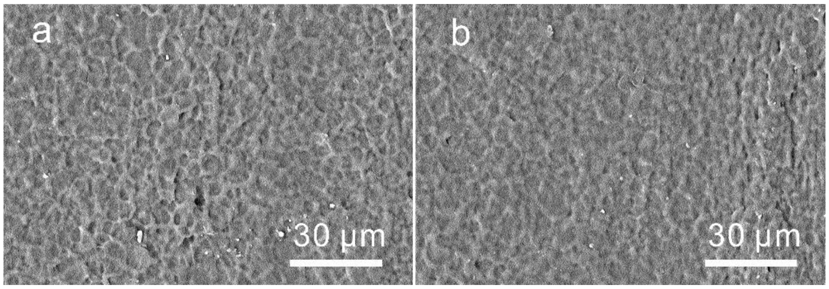

Supplementary Figure 6. a, b SEM images of ACEC@FEP and after immersion in water for 10 h.

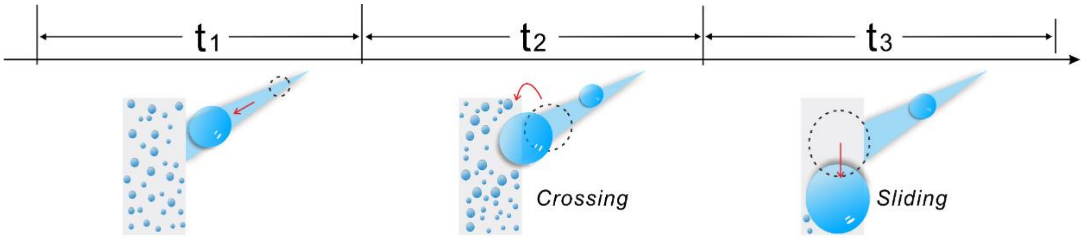

Supplementary Figure 7. Taking a single spine as an example, the droplet transmission during the harvesting is divided into three stages in detail: t_1: Time for water droplets creep from the spine tip to the root; t_2: Time for droplets grow and then cross into hydrophobic channels; t_3: Time for the droplet hangs at the junction until falls off.

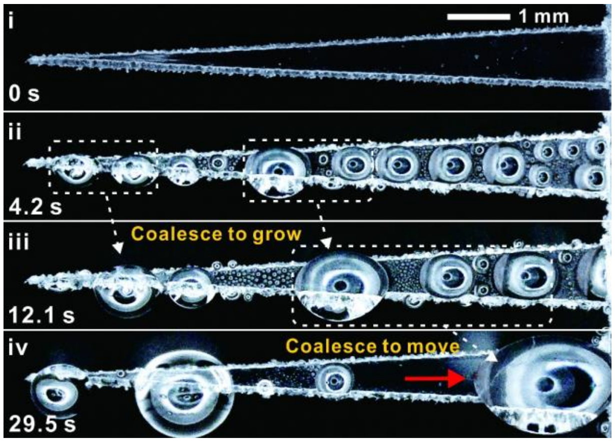

Supplementary Figure 8. Water collection process of the FEP spine in horizontal direction.

Supplementary Note 2. Water harvesting efficiency of asymmetric structures with different Laplace pressures

The Laplace pressure (*ΔP*) of the asymmetric surface can be calculated according to formula S3

$$\Delta P = \frac{dP}{dz}\Big|_{\Omega} = -\frac{2\gamma}{(r+R_0)^2}\sin\alpha \quad \text{(S3)}$$

where *Ω* is the droplet volume, *γ* is the surface tension of the droplet, *r* is the surface local radius, R_0 is the droplet radius, and *α* is the half vertex angle. It can be seen from the formula that *ΔP* increases as *sinα* decreased. Therefore, a small *sinα* contributes to a large *ΔP*. It can be seen from Fig. S9 that the water harvesting efficiency of asymmetric surfaces with different widths and heights increased with the decrease of *sinα*. It is worth noting that, due to limitations in instrument accuracy and material properties, the smallest sinα was obtained at a height of 10 mm and a width of 1 mm.

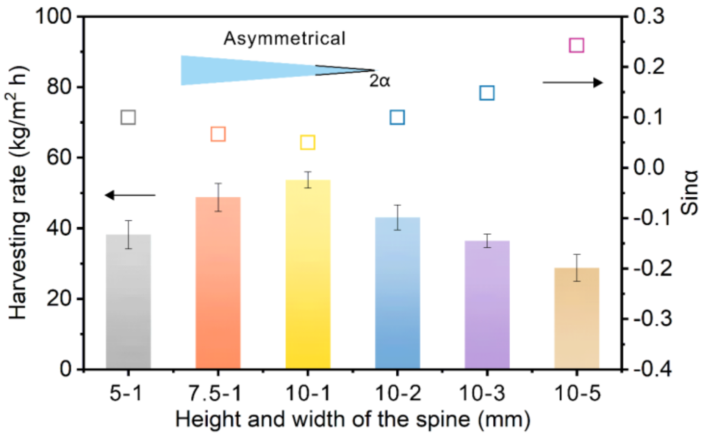

Supplementary Figure 9. The water harvesting efficiency and sinα of asymmetric/symmetric structures.

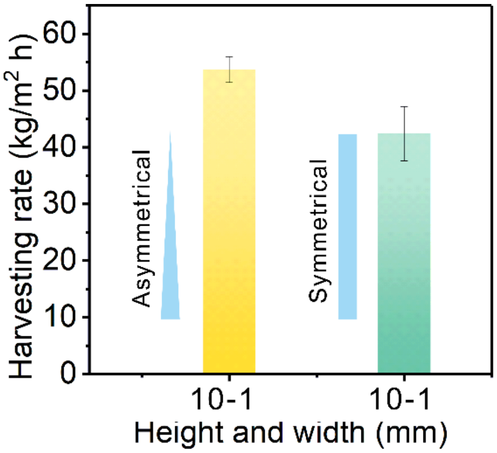

Supplementary Figure 10. The water harvesting efficiency of symmetric and asymmetric structure. Notably, the asymmetric was greater than that of the symmetric. Therefore, an asymmetric structure with a height of 10 mm and a width of 1 mm was chosen.

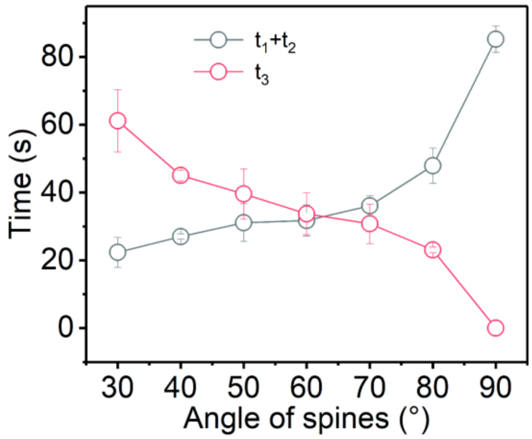

Supplementary Figure 11. During the water harvesting, t_1+t_2 increased as the angle increased, and t_3 decreased gradually as the angle increased.

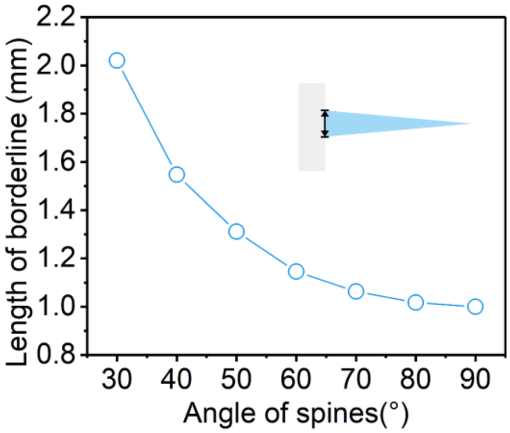

Supplementary Figure 12. The length of the borderline decreased from 2.02 mm to 1 mm with increasing angle (The distance between the midpoint of the borderline and the tip of the spine is 10 mm).

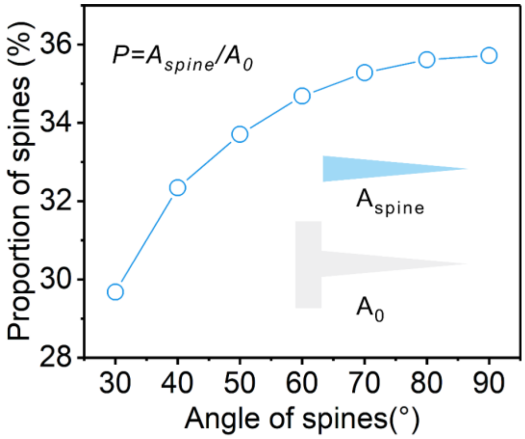

Supplementary Figure 13. The ratio of spine area corresponding to different inclination angles (The distance between the midpoint of the junction line and the tip of the spine is 10 mm).

Supplementary Note 3. Dynamic process of the droplet at different hydrophobic channel width

As can be observed, the merged droplets hung at the junction of the narrower hydrophobic channel due to excessive solid-liquid contact lines. As the width increased, the hanging time of the droplet decreased. Nevertheless, wider hydrophobic channels presented larger droplet coalescence distances, which would delay or hinder droplet coalescence. Therefore, a decrease or increase in width resulted in a delay in the droplet movement.

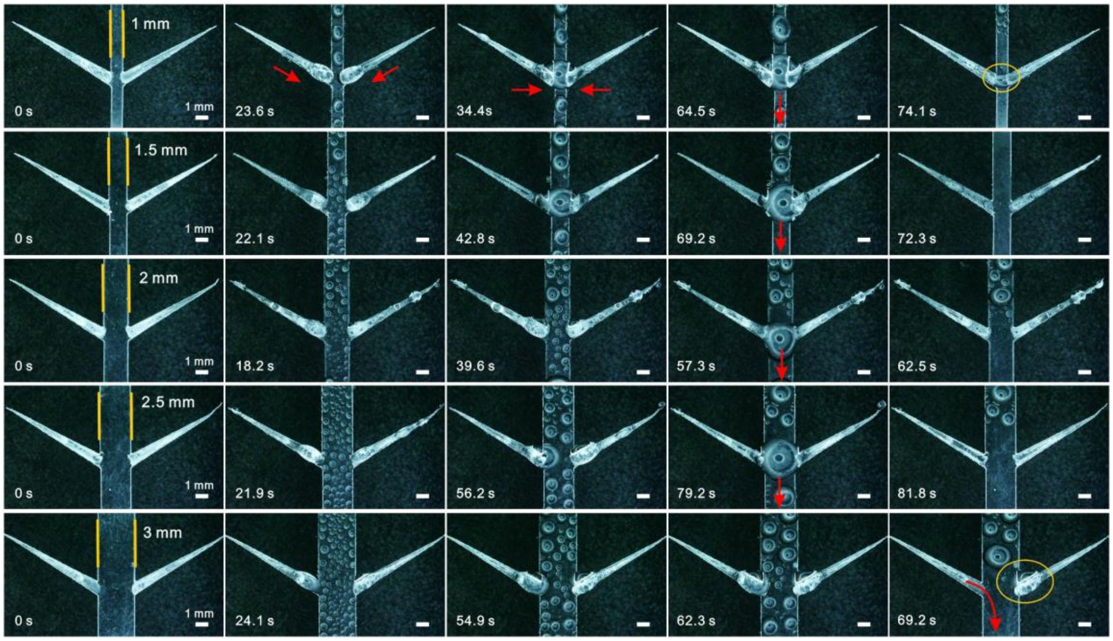

Supplementary Figure 14. The dynamic process of the droplet at different hydrophobic channel width (1-3 mm, tilt angle=60°).

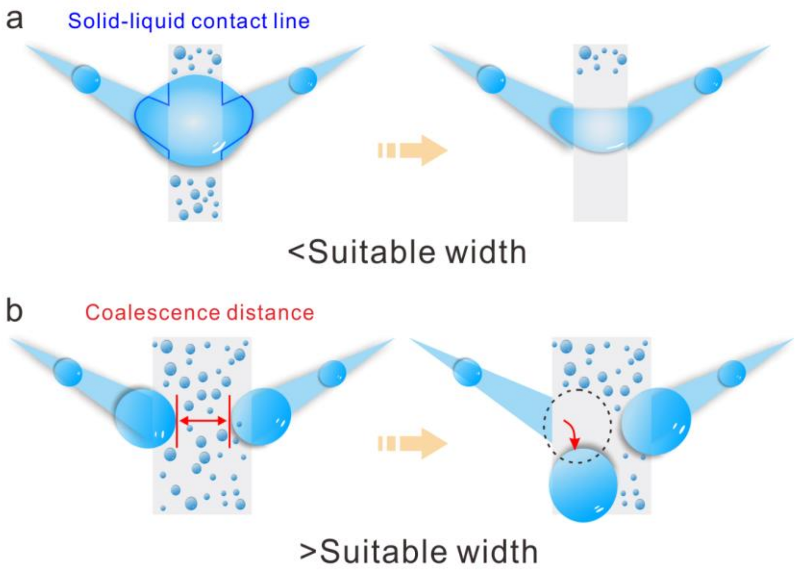

Supplementary Figure 15. Schematic diagram of fog harvesting with smaller or larger width of the transport channel.

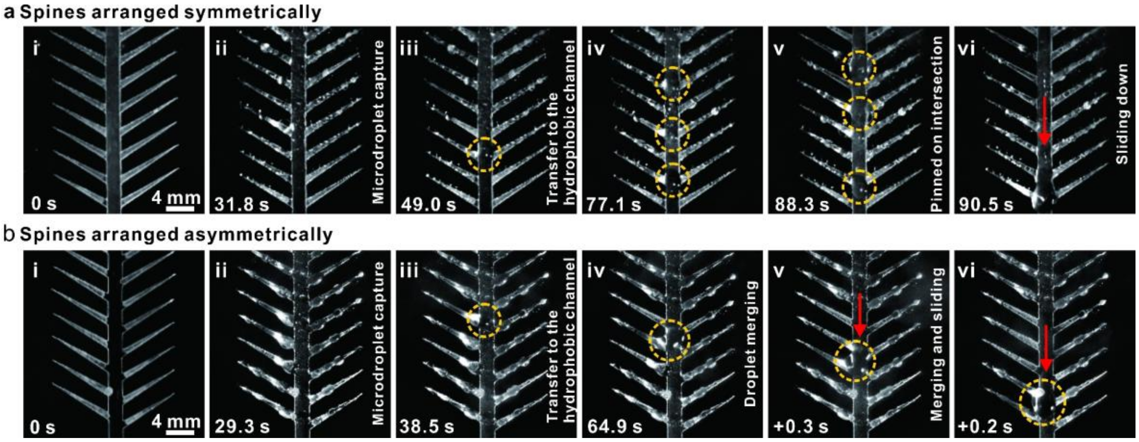

Supplementary Figure 16. Optical photographs of the fog collection process for (**a**) symmetric structures (ipsilateral and contralateral vertical distances were 2 and 0 mm, respectively) and (**b**) asymmetric structures (ipsilateral and contralateral vertical distances were 2 and 1.2 mm, respectively).

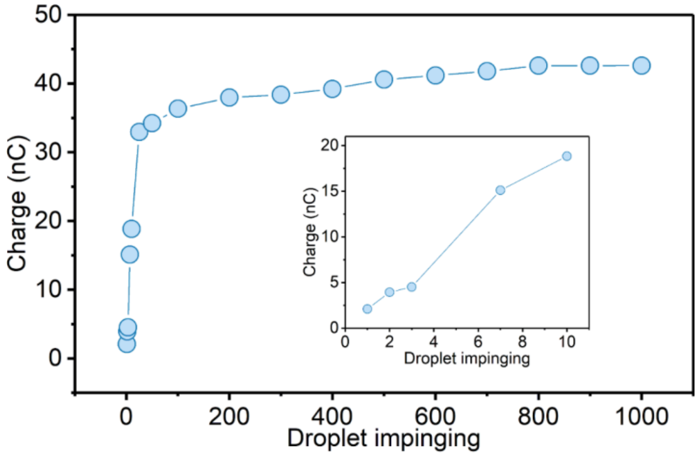

Supplementary Figure 17. As the droplets continue to impinge on the D-TENG, the amount of charge on the FEP surface increased gradually followed by remaining stable.

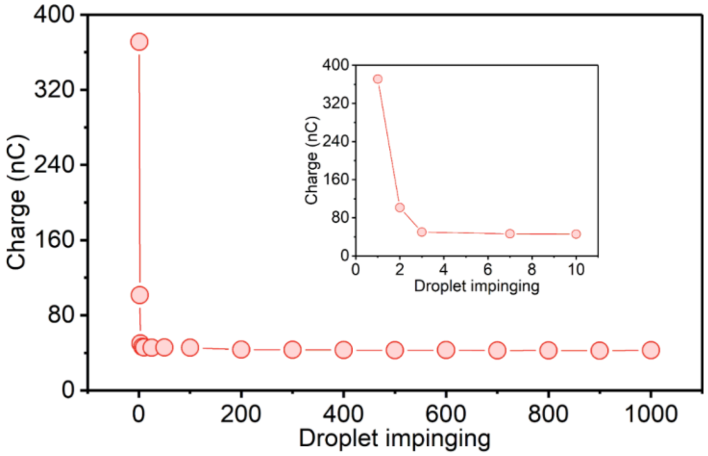

Supplementary Figure 18. After ion injection on the D-TENG surface, the transfer charge decreased rapidly from 371 nC followed by remaining stable.

Supplementary Note 4. Output performance of the D-TENG at different impacting angles

As the impact angle increased from 15° to 75°, the open-circuit voltage increased from 80.1 V to 103.2 V, followed by a gradual decreased to 51.6 V, and the corresponding transfer charge increased from 27.6 nC to 42.4 nC and then decreased to 17.9 nC. The highest value was shown at 45°, which is consistent with other work[1-2].

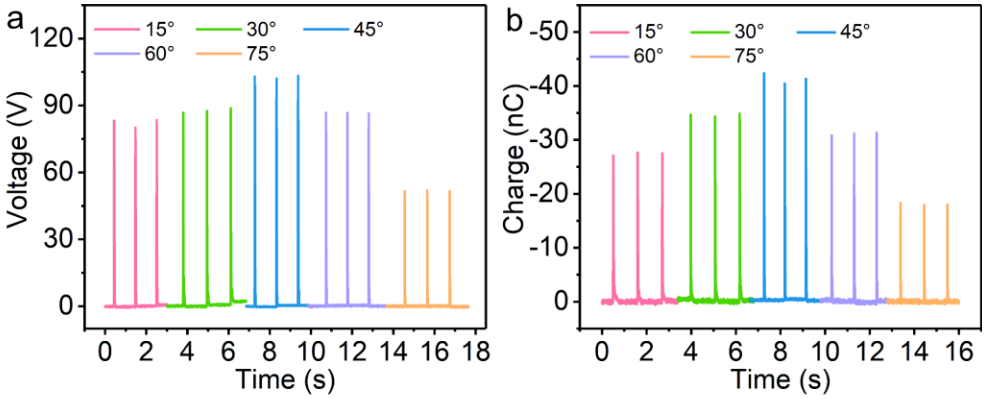

Supplementary Figure 19. Open-circuit voltage and transfer charge of the D-TENG at different impacting angles.

Supplementary Note 5. Output performance of the D-TENG for different droplet volumes

As the droplet volume increased from 54 μL to 96 μL, the open-circuit voltage increased from 61.2 V to 103.2 V, the transferred charge increased from 22.1 nC to 42.4 nC.

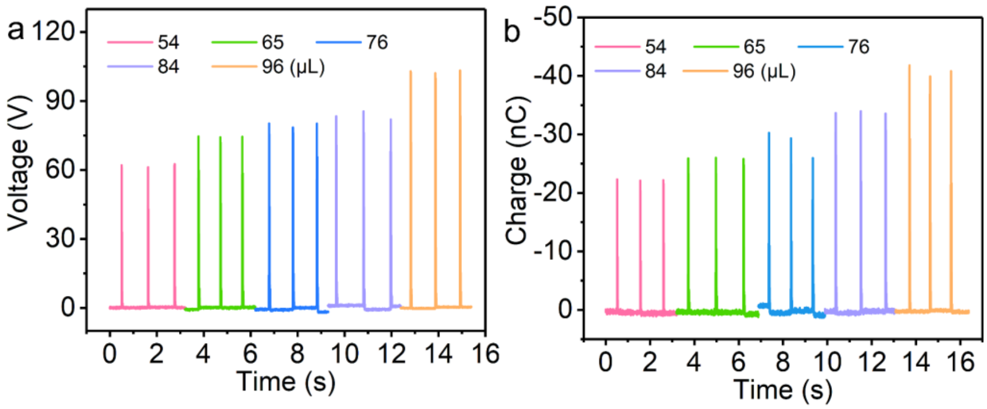

Supplementary Figure 20. Voltage and transferred charge of the D-TENG for different droplet volumes.

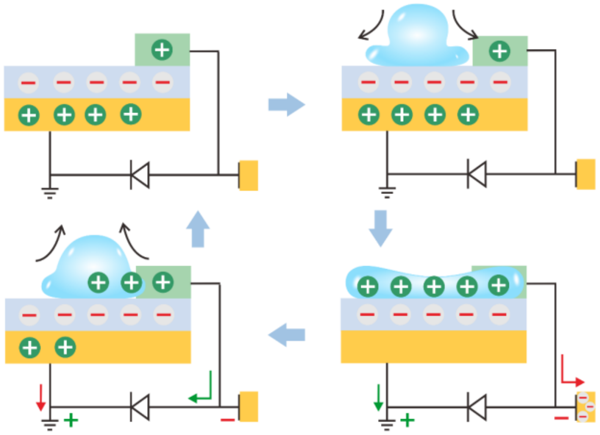

Supplementary Figure 21. Working mechanism of the negative charge generator.

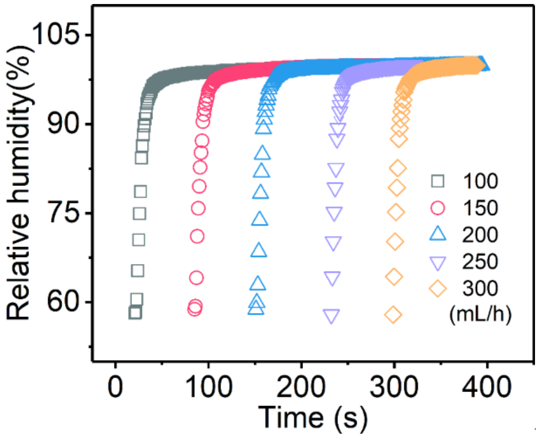

Supplementary Figure 22. As the fog flow rate increased from 100 mL/h to 100 mL/h, the relative humidity around the amphiphilic asymmetric surface could reach 99.9% successively (ambient temperature ~25°C, ambient humidity 58 ± 1%).

Supplementary Table 1. Comparison of recent studies on water harvesting

Bionic objects	Methods	Distance (cm)/Fog speed (cm/s) /humidity	water harvesting rate (kg/m^2 h)	References
Cactus	Coating zwitterionic carboxybetaine on antibacterial needle-array	5/70/85-90%	10.65	44
Cactus	Laser engraving followed by impregnation	5/2.2/NG	40.00	15
Beetle	Assemble copper mesh and cotton	15/70/NG	9.00	45
Beetle	Pulsed laser deposition with masks	NG	53.00	46
Beetle	In situ growth on the protein surface	NG/50/NG	91.7	47
Beetle	Spraying of micro-nanoparticles on substrate and subsequent modification	5/50/85-90%	17.07	48
Beetle	Selective modification	2/50/NG	22.00	49
Cactus and beetle	Photolithography and DRIE process	18/60-70/80	11.90	50
Cactus and beetle	Electrospinning and anodizing	NG	69.60	51
Cactus and beetle	Coating on the asymmetrical spines	5/30/99.9	93.18	This work

Supplementary Table 2. State of amphiphilic asymmetric surfaces at different wind velocity.

Wind velocity (m/s)	1.4	2.8	4.2	6.3	8.9	11.3	16.2
Condition	Good	Good	Good	Good	Good	Good	Good

Supplementary References

1. Wang L, *et al.* Harvesting energy from high-frequency impinging water droplets by a droplet-based electricity generator. *EcoMat.* 3:e12116 (2021).
2. Xu W, *et al.* A droplet-based electricity generator with high instantaneous power density. *Nature* **578**, 392-396 (2020).
