# Supplementary material for: Bioinspired asymmetric amphiphilic surface for triboelectric enhanced efficient water harvesting
Source: Nat Commun. 2022 Jul 18;13:4168. doi: 10.1038/s41467-022-31987-w (PMC9293931; doi:10.1038/s41467-022-31987-w)
Supplement: Supplementary file 3 — Description of Additional Supplementary Files [file 41467_2022_31987_MOESM3_ESM.pdf]

Description of Additional Supplementary Files

File Name: Supplementary Movie 1
Description: 400 commercial LEDs instantaneously powered by fog droplets impinging on device.

File Name: Supplementary Movie 2
Description: Effect of FEP on water mist before and after charging.
